# The Largest Operated Ascending Aortic Aneurysm in a Patient Without a History of Cardiac Surgery

**DOI:** 10.21470/1678-9741-2020-0052

**Published:** 2020

**Authors:** Engin Mesut, Sivri Ismail

**Affiliations:** 1 Department of Cardiovascular Surgery, University of Health Sciences, Mehmet Akif İnan Training and Research Hospital, Şanlıurfa, Turkey. E-mail: mesut_kvc_cor@hotmail.com; 2 Department of Anatomy, Kocaeli University School of Medicine, Kocaeli, Turkey.

**Dear Editor,**

We have read the case report by Bicer et al.^[1]^ entitled “The Largest Reported Giant Ascending Aortic Aneurysm Presented with Superior Vena Cava Syndrome”. First of all, we congratulate the authors for their invaluable contribution to the literature. On the other hand, we would like to clarify some points about the giant ascending aortic aneurysms.

Ascending aortic aneurysms are important diseases that require close clinical follow-up. When the diameter of aneurysm reaches to 6 cm, the risk of rupture approaches approximately 15%. In addition, as the diameter increases, the risk of dissection and rupture increases. The treatment option is urgent surgery when the acute dissection develops^[2]^. Factors such as hypertension and collagen tissue diseases can be effective in the development of aneurysm and dissection. In addition, it has been shown that some molecular tissue factors may play a role in the development of aortic dissection in recent studies^[3,4]^. As Bicer et al. point out, cystic medial necrosis is the most common cause of aneurysm. Furthermore, atherosclerotic degeneration, aortic valve diseases, and tissue diseases, such as Marfan syndrome, can cause this condition^[1]^. Accordingly, sharing the pathological diagnosis of this current case by the authors will contribute significantly to the literature. Also, it is not understood from the figures whether the thrombosed dissection line in the thrombosed dissected aortic aneurysm extends into the aortic arch. In addition, intraoperative evaluation on this subject was not elaborated by the authors. However, as far as we understand from the operation techniques, the operation was carried out by placing a single cross-clamp before the aortic arch. Although this is not clear, it suggests isolated thrombosed dissected ascending aortic aneurysm. Is this case an isolated thrombosed dissected ascending aneurysm?

The ascending aortic diameter of 10 cm and above is described as a giant aneurysm. In the literature, aneurysms which had larger dimensions than that of the current case have been reported. A case was reported several months ago with similar magnitude to this current case. An ascending aortic aneurysm of size 80 x 107 x 140 mm was detected in a 53-year-old female patient and was reported by Shah et al.^[5]^ However, this patient could not be operated because the patient did not accept the procedure. In 2009, a huge ascending aortic aneurysm was reported by Goncu et al.^[6]^ - an aneurysm reaching to 16 cm in diameter in a 28-year-old male patient. This patient, who underwent mechanical prosthetic aortic valve operation 10 years ago, underwent ascending aortic replacement accompanied by coronary button anostomosis. In this patient, Marfan syndrome was clinically excluded and the pathological diagnosis was detected as cystic medial necrosis. The patient was discharged on the 10^th^ day after the operation with healing^[6]^. In the light of all this information, it may be more accurate to describe this current case reported by Bicer et al.^[6]^ as “The largest operated ascending aortic aneurysm in a patient without a history of cardiac surgery”.
